# Elevated glucose acts directly on osteocytes to increase sclerostin expression in diabetes

**DOI:** 10.1038/s41598-019-52224-3

**Published:** 2019-11-22

**Authors:** Donna M. Pacicca, Tammy Brown, Dara Watkins, Karen Kover, Yun Yan, Matthew Prideaux, Lynda Bonewald

**Affiliations:** 1 Children’s Mercy Hospital, Kansas City, Missouri USA; 2 University of Missouri-Kansas City School of Dentistry, Kansas City, Missouri USA; 3 Indiana University, Indianapolis, Indiana, USA

**Keywords:** Diabetes complications, Diabetes complications

## Abstract

Bone quality in diabetic patients is compromised, leading to weaker bones and increased fracture risk. However, the mechanism by which this occurs in diabetic bone remains to be fully elucidated. We hypothesized that elevated glucose and glucose variation would affect the function of osteocytes, essential regulators of bone homeostasis and quality. To first test this hypothesis, we used the IDG-SW3 osteocyte-like cell line to examine the effects of glucose levels on osteocyte function and viability *in vitro*. We confirmed our *in vitro* findings using the *in vivo* streptozotocin-induced (STZ) diabetic rat model and *ex-vivo* cultured osteocytes from these rats. IDG-SW3 cells cultured under high glucose conditions displayed significantly increased *Sost* mRNA(100-fold) and sclerostin protein, a negative regulator of bone formation(5000-fold), compared to cells in control media. mRNA expression of osteoblast markers such as *Osx*, *Ocn* and *Col1a1* was unaffected by glucose. Factors associated with osteoclast activation were affected by glucose, with *Rankl* being upregulated by low glucose. *Opg* was also transiently upregulated by high glucose in mature IDG-SW3 cells. Induction of diabetes in Sprague-Dawley rats via a single dose of STZ (70 mg/kg) resulted in elevated maximum glucose and increased variability compared to control animals (670/796 vs. 102/142 mg/dL). This was accompanied by increased *Sost*/sclerostin expression in the osteocytes of these animals. These results show that glucose levels directly regulate osteocyte function through sclerostin expression and suggest a potential mechanism for the negative impact of diabetes on bone quality.

## Introduction

Diabetes mellitus is a disease with an increasing impact worldwide. Currently, an estimated 422 million people have diabetes^1^, and it has moved up to be the 6^th^ leading cause of death worldwide^2^. Fluctuations in blood glucose have a detrimental effect on heart, kidney, retinal and vascular tissues, and can lead to myocardial and cerebral infarction, kidney failure, blindness, neuropathy and infections that can result in amputation^3^. In addition, diabetic patients are known to have an increased risk for fracture that appears to be independent of bone mineral density. While type 1 diabetes patients generally have modest reductions in BMD, their relative risk for hip fracture is significantly higher than predicted^4^. In the type 2 patients, who generally have normal to elevated BMD, the relative risk for hip fractures is 1.7, also higher than expected or predicted^4^. While the fall risk for diabetic patients is also increased, this alone does not account for the increased fracture risk observed in these patients^5^.

Maintaining bone quality and integrity requires the coordinated actions of osteoblasts, which synthesize new bone matrix and osteoclasts, which resorb old or damaged bone. Osteocytes, cells which reside within the mineralized bone matrix, are responsible for regulating the activities of osteoblasts and osteoclasts in response to mechanical and biochemical signals^6,7^. Osteocytes have been shown to induce bone resorption via their production of the osteoclast-activating factor Receptor Activator of Nuclear factor Kappa-B Ligand (RANKL)^8,9^ and to inhibit bone formation by secreting sclerostin^10–12^, which is encoded by the *Sost* gene and is an important negative feedback regulator of the Wnt pathway^13,14^. Interestingly, serum sclerostin has been shown to be elevated in both type 1 and type 2 diabetes patients^15,16^. As sclerostin is primarily produced by osteocytes, this suggests that changes in glucose concentration may have a profound effect on the cells most responsible for maintaining bone health. More specifically, increased glucose variability as demonstrated by significant elevation and depression of blood glucose level well above and below the normal 80–140 mg/dL range may lead to adverse effects on osteocytes.

To investigate the role of glucose variability on osteocytes, we first used the IDG-SW3 cell line to examine the effects of varying glucose concentration on osteocytes *in vitro*. This cell line has been shown to differentiate from an osteoblast to an osteocyte-like phenotype during culture and, when fully differentiated, to recapitulate the phenotype of mature osteocytes *in vivo*^17^. In addition, we obtained serum and cortical bone specimens from normal and diabetic rats and isolated a purified primary osteocyte population to evaluate glucose effects on primary osteocyte function. We have used these *in vivo* and *in vitro* models to determine the effects of high glucose levels on osteocyte function and viability, which may have important implications for bone quality and susceptibility to fracture.

## Methods

### *In vitro* studies

#### IDG-SW3 cell line culture

The IDG-SW3 cell line was cultured as previously described^17^. Briefly, IDG-SW3 cells were expanded in permissive conditions (33 °C in alpha-MEM with 10% FBS, 100 U/ml penicillin, 50 µg/ml streptomycin, and 50 U/ml IFN-γ (Thermo Fisher Scientific)) on rat tail type I collagen-coated 150 cm^2^ culture dishes (Corning Inc.), then plated at 8 × 10^4^ cells/cm^2^ in osteogenic conditions (37 °C in DMEM (Mediatech Inc.) with 50 µg/ml ascorbic acid and 4 mM β-glycerophosphate (Sigma-Aldrich Corp., St. Louis, MO) under three different glucose concentrations: Low (2.5 mM equivalent to 45 mg/dl), Normal control (10 mM equivalent to 180 mg/dl), High (25 mM equivalent to 450 mg/dl); Mannitol control (glucose 10 mM with mannitol 15 mM (Sigma-Aldrich Corp., St. Louis, MO)) was used as a control for high osmolarity. Media was changed daily for 35 days. Cells were harvested at 3, 7, 14, 21, 28 and 35 days. There were three biological replicates for each of the conditions.

#### Measurement of metabolic activity *in vitro*

Media glucose concentrations in the IDG-SW3 cell cultures were obtained via glucometer (OneTouch Ultra 2, Lifescan, Milpitas, CA) from all wells at baseline (1 day pre-harvest) and at each harvest. These measurements were then used to calculate the amount of glucose utilized. Note that the lower limit of glucose measurement by glucometer is 20 mg/dL, with overall SEM of ±20% per manufacturer. As such, three measurements were obtained for each sample and averaged. We validated glucometer measurements of media with glucose at several different concentrations prior to initiation of experiments.

As previous studies have shown that bone primarily uses glycolysis for energy generation^18^, L-lactate assay (Eton Bioscience, San Diego, CA) was also performed on SW3 media per manufacturer’s instructions. Briefly, 50 µL L-lactate assay solution was added to a 96-well plate containing 50 µL standards and samples in duplicate, and incubated at 37°C for 30 minutes. The reaction was stopped with the addition of 0.5 M acetic acid and absorbance measured at 450 nm. The standards were used to interpolate lactate concentration.

We used LDH levels in cell lysate to estimate viability, with LDH activity in culture media to estimate cell death. LDH assay (Lactate dehydrogenase assay, Tox-7 kit, Sigma-Aldrich, St. Louis, MO) was performed on SW3 cell lysate as well as culture media per manufacturer’s instructions. Briefly, cells were lysed after a 50 µL sample of media was aliquoted. The lysed cells were centrifuged at 250 g for 4 minutes and the supernatant aliquoted. Samples were then placed into a 96-well plate with 100 µL of the assay mixture, covered and incubated at room temperature for 30 minutes. 1 N HCl was used to terminate the reaction. Absorbance of samples was read at 490 and 690 nm (Epoch BioTek plate reader, Winooski, VT).

#### Determination of relative cell number through DNA quantitation

IDG-SW3 cell cultures were normalized to approximate cell number using total DNA measurements, as mineralization did not allow for direct counting of differentiated cells. IDG-SW3 cells were grown for three days, then the cells were trypsinized, counted and placed into Trizol. Total DNA was isolated using the manufacturer’s protocol. Optical density was measured using a NanoDrop 2000 (Thermo Fisher Scientific, Wilmington, DE) to calculate concentration of DNA. This was related back to the counted cell number to determine the approximate DNA concentration per cell (9.2105E-4 pg/cell).

#### Assessment of matrix mineralization

IDG-SW3 cells were fixed with 10% formalin (Sigma-Aldrich, St. Louis, MO) for 5 minutes, washed with PBS, stained with 2% alizarin red S for 5 minutes, and repeatedly washed in PBS until no stain was present in solution. Plates were photographed, and then the dye was leached from the cultures using 500 µL 10% cetylpyridinium chloride solution at 37 °C for 30 minutes. Absorbance was measured at 570 nm.

#### Assessment of gene expression by real-time PCR

TRIzol reagent was used to isolate RNA: the digested bone chips were first placed in 1 ml TRIzol then pulverized in liquid nitrogen; the primary bone cell and IDG-SW3 cultures were scraped into 1 ml TRIzol. Total RNA was extracted using chloroform (Sigma) and purified using isopropanol (Sigma) according to the manufacturer’s instructions. RNA was washed in 70% ethanol and resuspended in RNase-free water. DNA contaminants were removed by digestion with DNase I (DNA-free™ DNA Removal Kit, Applied Biosystems, Carlsbad, CA) for 30 minutes at 37 °C. 2–4 µg of RNA were reverse transcribed to cDNA using the High Capacity cDNA Reverse Transcription Kit (Applied Biosystems, Foster City, CA) according to the manufacturer’s instructions. Real-time PCR was performed on a Step One Plus cycler using 25 ng of template cDNA with 2x Taqman Gene Expression Master Mix and Taqman gene arrays for *Sost*, *Actb*, *Ocn*, *Opg*, *Rankl*, *E11*, *Dmp1*, *Glut1*, *Glut2*, *Glut3* and *Glut4* (all from Applied Biosystems, Carlsbad, CA).

Gene expression was normalized to the housekeeping gene *Actb* and relative quantification was calculated with the 2^−ΔΔ^*C*T method^19^ using the normal glucose as control for low glucose, with the mannitol plus glucose as control for high glucose. Normal glucose vs. mannitol was also examined to evaluate for any effect of osmolarity.

### *In vivo* studies

N.B.: the tissues and data collected for this study were taken from animals already approved for experiments in the laboratories of Drs. Kover and Yan, therefore maximizing tissue usage and reducing animal wastage. The University of Missouri at Kansas City (UMKC) animal facility is operated as a specific pathogen-free, Association for Assessment and Accreditation of Laboratory Animal Care (AAALAC)-approved facility and animal care and husbandry meet the requirements in the Guide for the Care and use of Laboratory Animals (8th edition), National Research Council. All animal experiments were approved by the UMKC Institutional Animal Care and Use Committee (IACUC) in accordance with relevant federal regulations and guidelines (protocols 1229, 1603).

#### Induction of diabetes *in vivo*

In Drs. Kover and Yan’s laboratory, chemical diabetes was induced in randomly allocated 8 week old healthy male Sprague-Dawley rats (Harlan Sprague Dawley Inc, Indianapolis, IN) via a single dose of intraperitoneal streptozotocin (70 mg/kg, Sigma-Aldrich Corp., St. Louis, MO) versus normal saline for controls. Induction of diabetes was confirmed by 48 hours in all diabetic animals via tailstick glucose measurement. A subcutaneous osmotic insulin pump (DURECT Corporation, Cupertino, CA) was inserted into the diabetic rats at day 8 providing insulin-aspart (Novolog^®^) 1U/kg/day (Novo Nordisk, Plainsboro, NJ) which was used to maintain a controlled level of hyperglycemia (per lab protocol). Animals were housed individually with free access to food and water. Water intake and weight were monitored. Tailstick blood glucose measurements were obtained daily in diabetic and selected control animals. In addition, selected rats from both groups had continuous glucose monitoring (q 5 minute) via a subcutaneous sensor (Medtronic, Northridge, CA) for three days. Rats were sacrificed at 8 weeks post injection. No adverse events were noted.

#### Isolation of primary rat osteocytes

Long bones (femur, tibia or humerus) were harvested from rats and soft tissue was mechanically removed from the surface of the bone. A bone cutter was used to split the bone in half and remove the marrow, rinsing in sterile Dulbecco’s phosphate buffered saline (PBS, Mediatech, Inc. Manassas, VA) simultaneously. Once the soft tissue and marrow were removed, the bones were minced into 1–2 mm chips, washed in PBS and placed into α-minimum essential medium (α-MEM, Mediatech, Inc. Manassas, VA) with 1% penicillin/streptomycin (P/S, 10,000 U/mL, 15140122, Invitrogen, Carlsbad, CA). The chips were put through six cycles of alternating 1 mg/mL collagenase digests (Sigma-Aldrich Corp., St. Louis, MO) and 14 mM ethylene diaminetetraacetic acid (EDTA, Sigma-Aldrich Corp., St. Louis, MO) with 1% bovine serum albumin (BSA, Sigma-Aldrich Corp, St. Louis, MO) in an incubator set at 37 °C for 20 minutes to remove tissue and mineral. After each cycle, the chips were centrifuged at 400 g for 5 minutes with PBS. Following the 6 cycles, the bone chips were used for culture or downstream RNA analysis.

#### Primary osteocyte-enriched culture

An osteocyte-enriched population of cells was isolated from the long bones of the 16 week old rats as described previously. Cells were obtained from the supernatants of digests 5 and 6, centrifuged at 400 g for 5 minutes and re-suspended in 3 mL of culture media (α-MEM supplemented with 1% P/S, 5% fetal bovine serum (FBS, Hyclone Laboratories, Logan, UT), 5% calf serum (CS, Hyclone Laboratories, Logan, UT)). These cells, with a portion of the bone chips from each animal, were then cultured for 72 hours in 6-well collagen-coated plates (Collagen I, rat tail, Fisher Scientific, Hampton, NH) in a total of 1.5 mL of culture media. After 72 hours, 1 mL of culture media was added to each well. At day 5 of culture, the media was replaced with 2 mL fresh culture media containing either 25 mM glucose or 5.5 mM glucose with 19.5 mM mannitol (Sigma-Aldrich Corp., St. Louis, MO). At day 7, media, cells and chips were harvested. Cells and chips were placed in 1 mL of Tri-Reagent (Molecular Research Center, Inc. Cincinnati, OH), flash frozen in liquid nitrogen, and stored at −80 °C to be used for RNA isolation. Conditioned media was flash frozen in liquid nitrogen, and stored at −80 °C to be used for assays. There were three biological replicates for each of the conditions.

#### Measurement of sclerostin protein *ex vivo* and *in vitro*

Sclerostin ELISA assay (R&D Systems, Minneapolis, MN) was performed on SW3 media harvested at days 21, 28 and 35 of culture, rat serum and rat primary bone cell media per manufacturer’s instructions. Briefly, 50 µL diluent solution was added to a conjugated 96-well plate containing 50 µL standards and samples in duplicate, and incubated at 37 °C for 3 hours. 100 µL of Mouse SOST conjugate was added per well and incubated at 37 °C for 1 hour. 100 µL of substrate solution was added per well and incubated in the dark at 37 °C for 30 minutes. The reaction was stopped with the addition of 100 µL of stop solution and absorbance measured at 450 nm, with readings at 540 nm obtained for wavelength correction. Standards were used to interpolate sclerostin concentration.

#### Immunohistochemistry

Digested bone chips (from random segments of bone) were fixed in 10% formalin for 24 hours and then decalcified for two weeks in 14% Ethylenediaminetetraacetic acid (EDTA, Sigma). The bone chips were processed through graded ethanol and xylene, paraffin-embedded and 6 µm sections were cut onto Superfrost Plus slides (Thermo Fisher Scientific). Sections were dewaxed in Clear-rite (Thermo Fisher Scientific, Kalamazoo, MI) and rehydrated prior to immunostaining. Sections were then incubated 1 hour at room temperature in blocking solution (0.2% gelatin (Knox, Winona, ON Canada); 0.5% nonfat dry milk (Wal-Mart, Bentonville, AR), 0.02% Tween-20 (Sigma), 1% serum of secondary antibody), followed by goat anti-sclerostin antibody (1:250, R&D Systems Inc., Minneapolis, MN) (1:500) overnight at 4 °C. Rabbit anti-goat antibody (1:200, Vector ABC kit, Vector, Burlingame, CA) was then incubated per kit instructions for 1 hour at room temperature for secondary antibody and avidin-biotin complex reaction, followed by DAB exposure for 1.5 minutes. Slides were counterstained with methyl green, rinsed and cover-slipped and imaged at 100x magnification. Cell counting was performed as an average of 6 images under 40x magnification, with percent positive cells expressed as number of positively stained cells over the total number of methyl green stained cells times 100. At least two slides were used for each specimen.

#### Fluorescent Immunohistochemistry

Whole bones (tibia) from control and diabetic rats were fixed in 10% formalin for 24 hours, then decalcified for 2–3 weeks in 14% EDTA. Bone sections were prepared as described above. These were dewaxed and rehydrated prior to immunostaining. Sections were then incubated in blocking solution, followed by goat anti-sclerostin antibody (1:250, R&D Systems Inc., Minneapolis, MN) overnight at 4 °C. Cy3-donkey anti-goat antibody (Jackson ImmunoResearch, Burlingame, CA) was then added after rinsing slides and incubated for 1 hour at room temperature. Slides were incubated with DAPI (Sigma) for 30 minutes at room temperature, rinsed and cover-slipped and imaged with a laser confocal microscope (Carl Zeiss Microscopy GmbH, Munich, Germany). Cell counting was performed as an average of 9 images of diaphyseal bone obtained from standardized regions (proximal, middle and distal regions from each cortex) under 25x oil magnification in blinded fashion. Percent positive cells were expressed as number of positively stained cells over the total number of DAPI-positive cells times 100. A minimum of two sections per bone were used for each specimen.

### Statistical analysis

Statistical analysis was performed using Prism 6 software (GraphPad, San Diego, CA). Linear and non-linear curve regressions were used for assay interpolation, using R squared value greater than 0.99 for goodness of fit. One- and two-way ANOVA with Tukey post hoc test were used to determine significant differences compared to controls, with *p* < 0.05 being considered significant. Data shown are mean ± SEM.

## Results

### Cell culture

#### High glucose does not affect osteocyte viability *in vitro*

**Figure 1:**
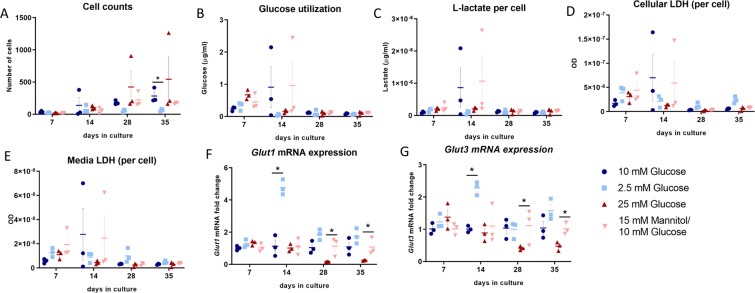
Elevated glucose levels do not affect cell number or activity of IDG-SW3 cells but regulate the expression of glucose transporter enzymes. (**A**) No change in approximate cell number was observed when culturing IDG-SW3 cells under high glucose conditions (as measured by total DNA content), but cell number was significantly decreased under low conditions by day 35 (p = 0.022). (**B**) No change in glucose utilization is observed when normalized to cell number. (**C**) Lactate production is not affected by glucose levels in IDG-SW3. Lactate dehydrogenase activity in cell lysate (**D**) or culture media (**E**) is unaffected by glucose. The transporter enzymes *Glut1* (**F**) and *Glut3* (**G**) are upregulated by low glucose and downregulated by high glucose culture conditions (*Glut 1* Ct^Low^ 22–27, Ct^High^ 25–27 vs. Ct^Normal^ 22–27; *Glut 3* Ct^Low^ 26–28, Ct^High^ 26–30 vs. Ct^Normal^ 26–28, p < 0.009, n = 3). Graphs show individual data points, mean and SEM for fold change in gene expression compared to control. 10 mM Glucose = normal levels, 2.5 mM Glucose = Low Glucose, 25 mM Glucose = High Glucose, 15 mM Mannitol/10 mM Glucose = Control for Osmolarity.

To interrogate the role of glucose in regulating osteocyte function and sclerostin *in vitro*, we first utilized the osteocyte-like cell line IDG-SW3. Long-term culture of IDG-SW3 cells under high glucose conditions did not affect the cell number; culturing the cells under low glucose conditions did significantly reduce the cell number after day 28 (Fig. 1A).

The data were then normalized to the estimated cell number (obtained indirectly via total DNA) to account for differences in cell density when cultured under different glucose levels. No differences in glucose utilization under the different culture conditions were observed when normalized to cell number (Fig. 1B). Similarly, no differences were observed in production of the glucose metabolite, lactate, by the IDG-SW3 cells under high or low glucose culture conditions (Fig. 1C).

Lactate dehydrogenase (LDH) assays were performed on both cell lysate and media, with lysate measurement reflecting live cell activity and media LDH proportional to numbers of dead cells. There were no significant differences observed in LDH levels in the cell lysate when corrected for cell number, indicating no differences in cell viability between glucose conditions (Fig. 1D). No decrease in LDH in conditioned media was noted with either high or low glucose (Fig. 1E). This suggests that high or low glucose culture conditions do not induce cell death in the IDG-SW3 cells. The differences between the earlier and later time points likely reflect decreased cytoplasmic volume of the more mature osteocytic cells.

#### Local glucose concentrations regulate glucose transporter enzyme expression in osteocytes

The glucose transporter enzymes play essential roles in the transportation of glucose across the plasma membrane. The expression of the glucose transporters *Glut1*-*Glut4* was assessed in IDG-SW3 cells by real-time PCR. *Glut1* mRNA expression was higher than *Glut3*, whereas *Glut2* and *Glut4* were not detectable (data not shown) in the IDG-SW3 cells. The expression of both *Glut1* and *Glut3* mRNA was significantly upregulated in culture under low glucose conditions and inhibited by high glucose (Fig. 1F,G). Insulin receptor expression was not affected by glucose concentration (Supplemental Fig. 1).

#### High glucose culture conditions promote sclerostin expression in osteocytes *in vitro*

**Figure 2:**
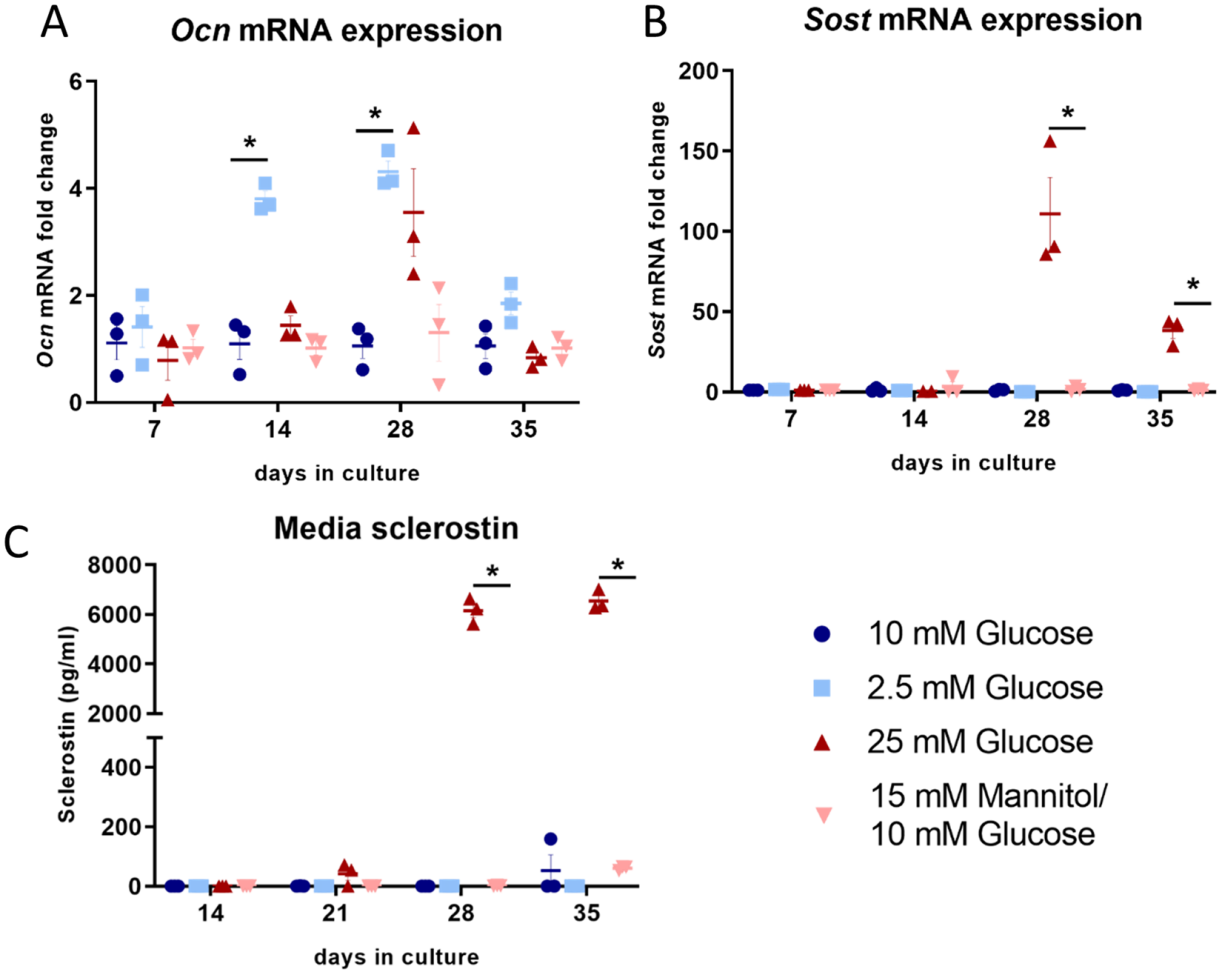
Sclerostin is upregulated by elevated glucose in IDG-SW3 cells. (**A**) *Ocn* mRNA shows a transient significant upregulation in response to low glucose (Ct^Low^ 24–27, Ct^High^ 26–28 vs. Ct^Normal^ 26–28). (**B**) *Sost* mRNA is strongly upregulated in response to high glucose in mature IDG-SW3 cells (Ct^High^ 23–30 vs. Ct^Normal^ 25–33, p < 0.0001, n = 3), as is (**C**) sclerostin protein (p < 0.0001, n = 3).

IDG-SW3 cells were cultured under low and high glucose conditions to evaluate the effects on bone-specific genes. *Ocn* expression was temporally upregulated in the mature IDG-SW3 cells under low glucose conditions (Fig. 2A). Most notably, high glucose significantly and robustly increased *Sost* mRNA expression in the mature, osteocyte-like IDG-SW3 cultures compared to mannitol control 50–150 fold (Fig. 2B). The increase in *Sost* mRNA was accompanied by an increase in sclerostin production by the cells cultured under high glucose 6000 fold (Fig. 2C).

#### Glucose levels do not affect mineralization *in vitro* but regulate markers associated with bone formation and resorption

**Figure 3:**
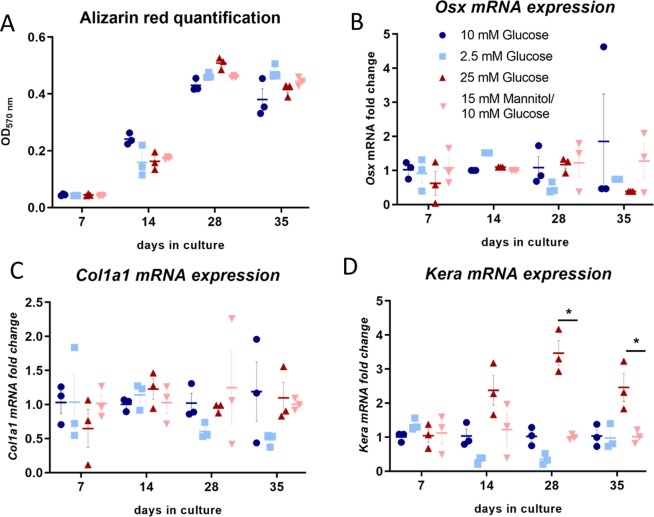
Effects of varying glucose concentration on matrix mineralization and expression of bone regulatory genes. (**A**) Matrix mineralization by IDG-SW3 cells is unaffected by glucose concentration. (**B**) *Osx* and (**C**) *Col1a1* mRNA expression is not regulated by high glucose. (**D**) *Kera* mRNA, a marker of osteoblast differentiation, is upregulated under high glucose culture conditions.

As sclerostin is known to regulate bone formation and mineralization^10,20^, we examined the mineral deposition and osteoblast marker expression of IDG-SW3 cells cultured under high and low glucose. Maximal mineralization of the cells was reached at day 21 and was unaffected by either high or low glucose (Fig. 3A). The expression of the osteoblast marker genes osterix (*Osx*) and *Col1a1*, was unaffected by glucose (Fig. 3B,C), as were the osteocyte marker genes *Dmp1* and *E11* (Supplemental Data).

### Animal studies

#### Streptozoticin injection induces high glucose levels *in vivo*

**Figure 4:**
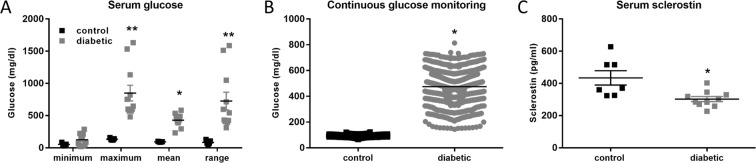
Analysis of glucose levels and serum sclerostin in diabetic animals. Blood glucose levels from tailstick (**A**) and 72-hour continuous subcutaneous monitoring (**B**) demonstrating significant elevation in blood glucose mean, maximum and range. (p < 0.0001, n = 11 diabetic, 5 control). (**C**) Serum ELISA results from normal vs. diabetic (p = 0.006, n = 8 diabetic, 7 control).

Tail stick and continuous glucose monitoring (CGMS) glucose measurements were taken from 5 control and 11 diabetic rats to determine the glucose levels resulting from inducing diabetes in these animals. There were significant differences in mean and maximum glucose as well as glucose range, which is a marker of variability (Fig. 4A). Glucose variability and maximum glucose were significantly increased in the diabetic vs. control animals (670/796 vs. 102/142 mg/dL) with CGM (Fig. 4B). No adverse events were noted. Unexpectedly, serum sclerostin levels were significantly lower in the diabetic animals compared with control rats (Fig. 4C).

#### Diabetes upregulates sclerostin expression in osteocytes *in vivo*

**Figure 5:**
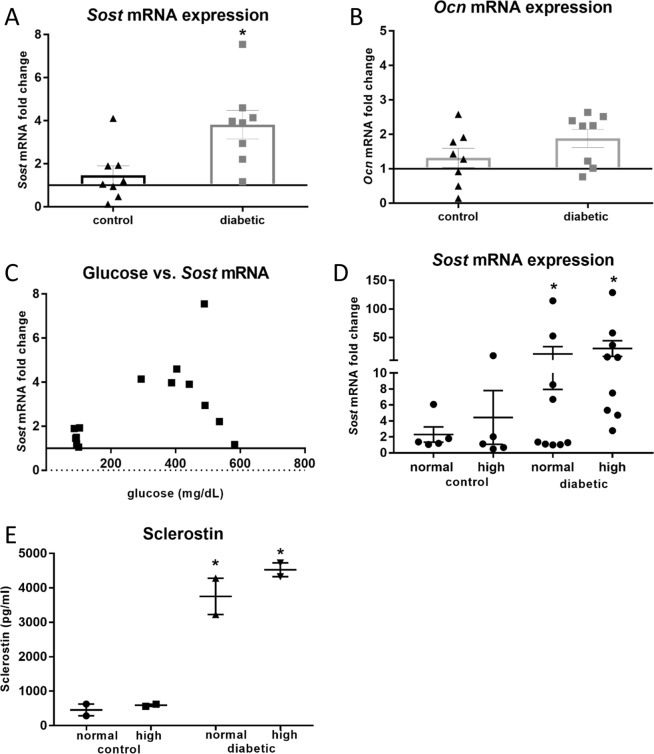
*Sost* expression is increased in diabetic bone. (**A**) *Sost* expression is increased almost 4-fold in diabetic bone (Ct^diabetes^ 30 vs. Ct^control^ 32) but (**B**) *Ocn* mRNA expression is unaffected. (p = 0.012, n = 8 diabetic, 8 control). (**C**) Sost expression correlated with glucose expression (p = 0.06, n = 8 diabetic, 6 control). Primary osteocyte culture from control and diabetic animals showed maintenance of *Sost* mRNA (**D**) and sclerostin protein expression (**E**) at significantly high levels independent of media glucose concentration (p < 0.0001 for both (**D**) and (**E**), n = 2 for each group, 3 replicates per treatment).

To determine the effects of glucose on Sost expression in osteocytes *in vivo*, we analyzed RNA from cortical bone from control and diabetic rats, which had been sequentially digested to remove cells from the bone surface. To confirm that the digested bone contained primarily osteocytes, real-time PCR demonstrated undetectable expression of the gene *Kera*, coding for the osteoblast marker keratocan^21^ (data not shown). Increased expression of *Sost* was observed in the digested bone from diabetic rats compared to controls (Fig. 5A), in contrast with the decreased levels of sclerostin in the serum. No difference was observed *Ocn* mRNA expression (Fig. 5B).

To examine the response of osteocyte populations from diabetic and control rats to high glucose, primary osteocyte-enriched cells and bone chips were isolated from rat cortical bone and cultured under normal and high glucose conditions. Osteocytes from the diabetic rats expressed high levels of *Sost* mRNA and sclerostin compared to those from the control rats (Fig. 5D,E). This was consistent across animals. This was observed despite culturing cells in normal media. Culturing the diabetic osteocytes in high glucose for 7 days did not significantly change expression. There was a trend towards a significant correlation between *Sost* mRNA and glucose taken from the animals (p = 0.06) (Fig. 5C).

**Figure 6:**
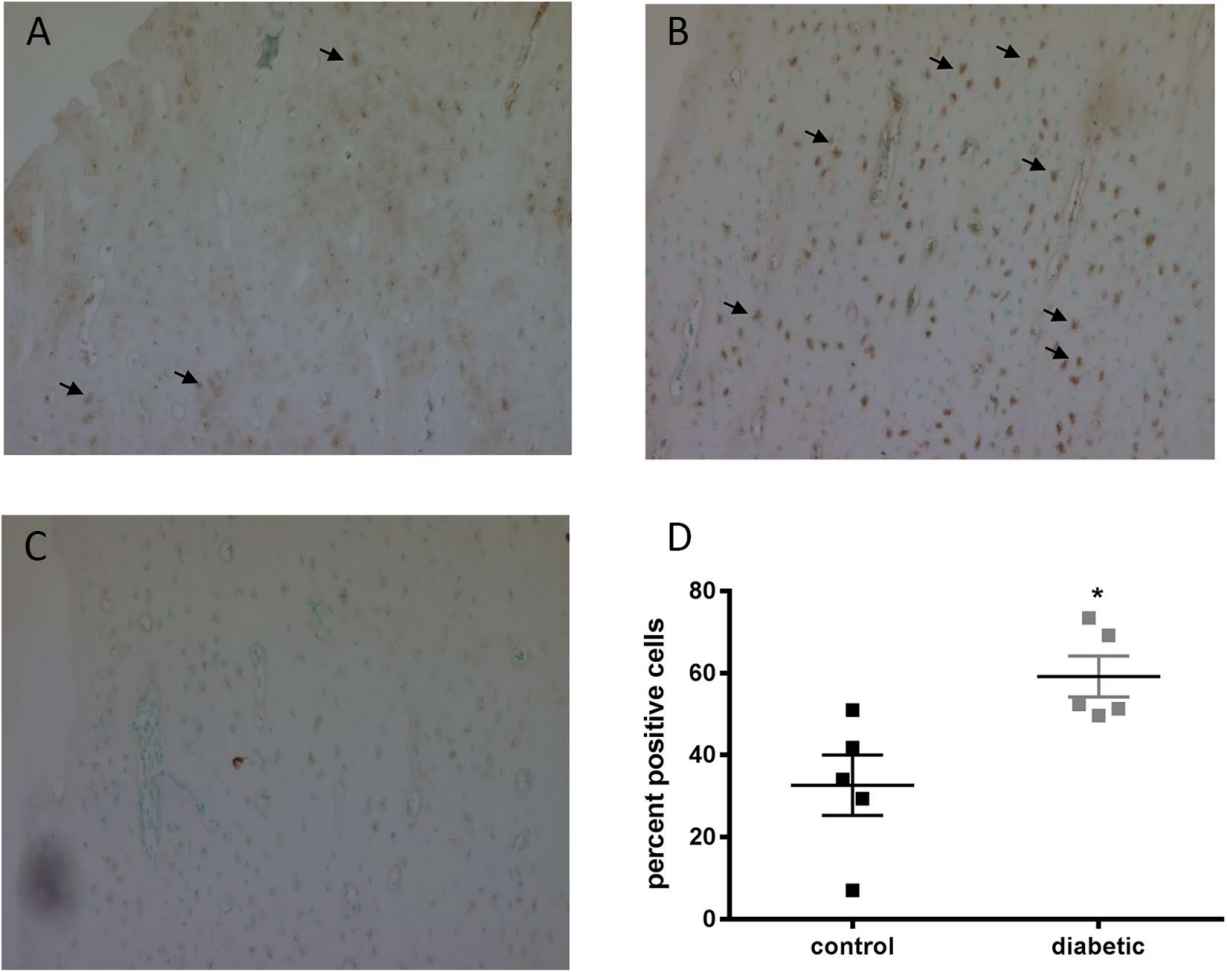
Sclerostin protein expression is elevated in bone chips from diabetic animals. Immunohistochemical staining for sclerostin in bone chips from cortical bone show increased sclerostin expression in diabetic bone (**B**) compared to control bone. (**A**) Non-immune control is shown in. (**C**) Sclerostin positive osteocytes are indicated by black arrows. (**D**) The percentage of sclerostin positive osteocytes is significantly increased in the diabetic bone pieces (p = 0.021, n = 5 per group).

**Figure 7:**
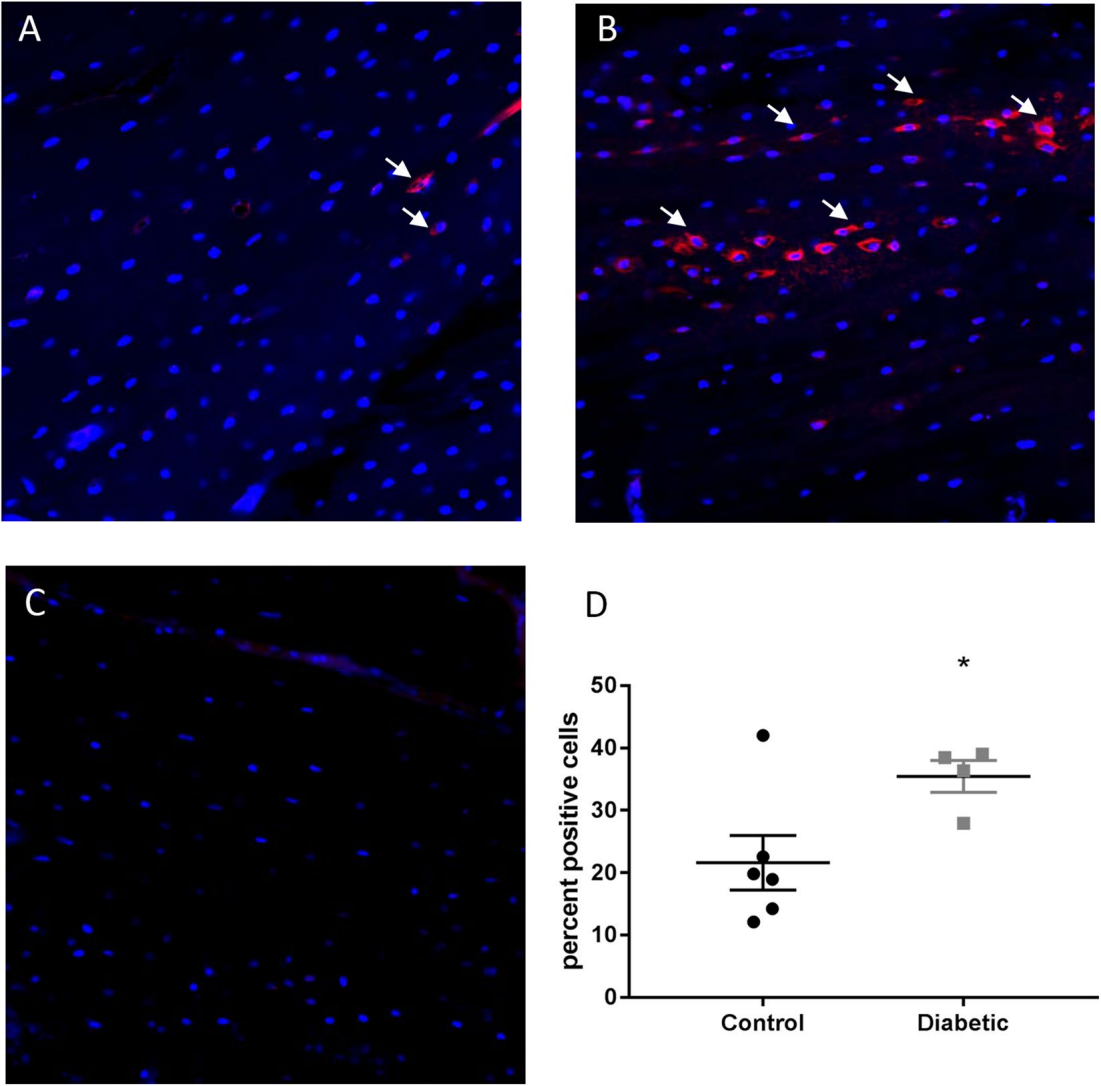
Sclerostin protein expression is increased in the tibiae of diabetic rats. Immunofluorescence staining for sclerostin in cortical bone shows increased sclerostin positive osteocytes (red staining, white arrows) in the tibiae from diabetic animals (**B**) compared to control. (**A**) Non-immune control is shown in. (**C**) The percentage of sclerostin positive osteocytes is significantly increased in the diabetic bone (p = 0.027, n = 4 diabetic, 6 control).

To evaluate whether the increased *Sost* mRNA expression in the cortical bone from the diabetic rats corresponds to increased sclerostin production, bone from the rats were assessed for sclerostin protein by immunohistochemistry (for the digested bone chips) as well as by confocal using immunofluorescence (for whole bone sections). Increased intensity of sclerostin staining was observed in the cortical bone chips from the diabetic rats compared to controls (Fig. 6A,B). Quantification of the percentage of positively stained cells demonstrated a significant increase in the diabetic rat bone chips vs the controls (Fig. 6D). This was confirmed by whole-bone immunofluorescence (Fig. 7). We noted that sclerostin expression was not uniform throughout the bone, but was randomly clustered in the cortex. This expression pattern did not appear to consistently localize to any one region (proximal vs. mid vs. distal cortex) across specimens.

## Discussion

Type 1 and type 2 diabetes are both associated with poor bone quality and increased risk of fracture, resulting in a lower quality of life for patients and an increased burden on the healthcare system^3,22^. Sclerostin, a known inhibitor of bone formation, has been shown to be upregulated in the serum of patients suffering from type 1 and type 2 diabetes^16,23,24^. In addition, sclerostin levels can be regulated by other complications of diabetes such as chronic kidney disease^25^ and inflammation^26^. Therefore, we investigated the effects of varying glucose levels in osteocytes *in vitro* to determine the direct effect of chronically elevated glucose levels on osteocytes, as well as confirming our findings with evaluation of streptozotocin-induced diabetes on sclerostin expression *in vivo* and *ex vivo*.

Dramatic elevation of *Sost* mRNA over 100 fold and sclerostin secretion over 6000 fold was observed in IDG-SW3 osteocyte-like cells cultured long-term under high glucose conditions, offering evidence that the upregulated sclerostin production by osteocytes in diabetes is mediated by local glucose levels. High glucose has also been shown to increase sclerostin in the MLO-Y4 cell line approximately 2 fold^27^. As the MLO-Y4 cells have been proposed to be a model of early osteocytes^28^ and as the IDG-SW3 represent a model of late osteoblast to early osteocyte to late osteocyte^17^, the 6000 fold increase in the late osteocyte stage suggests a greater sensitivity to glucose than late osteoblasts or early osteocytes. Given the dramatic increases in sclerostin observed, we next examined the effects on matrix mineralization and bone formation markers in IDG-SW3 cells cultured under high and low glucose conditions. No changes in expression of the osteoblast marker genes osterix (*Osx*) and type I collagen (*Col1a1*) were observed in response to low or high glucose. Interestingly, increased expression of the late osteoblast marker gene *Kera* was observed in the cells cultured under high glucose at the later time points. Although the protein product of *Kera*, keratocan, is thought to play a role in matrix mineralization^21^, no differences were observed in mineral deposition under differing glucose conditions as demonstrated by alizarin red staining. Therefore, the function of the increased *Kera* expression under high glucose conditions is currently unknown and will require further investigation.

Induction of high glucose levels *in vivo* also resulted in elevated *Sost* mRNA and robust sclerostin protein expression by primary osteocytes. However, serum sclerostin levels were significantly decreased in the diabetic animals, suggesting a discord between local and serum sclerostin expression. This therefore suggests that the increased sclerostin produced by the osteocytes is being retained within the bone. This suggests that measuring serum sclerostin in diabetic patients may not be the most reliable method in evaluating biological sclerostin activity and bone quality. It has recently been shown that in chronic kidney disease (CKD) patients treated with glucocorticoids, serum sclerostin levels are not associated with bone sclerostin, as assessed by immunohistochemistry^29^. Furthermore, similar to our observations in diabetic animals, bone *SOST* mRNA and sclerostin protein levels are increased in patients following a kidney transplant, but serum sclerostin levels are decreased^30^.

**Figure 8:**
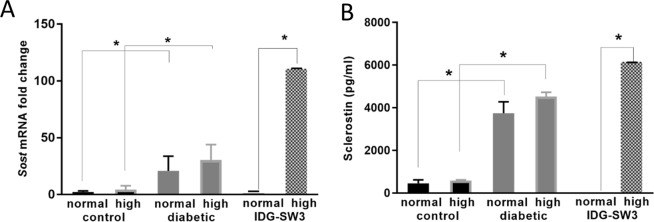
*Sost* mRNA (**A**) and sclerostin protein (**B**) are similarly regulated by chronic high glucose in both primary osteocytes and IDG-SW3 cells (n = 3, p < 0.0001). However, the primary osteocytes from animals exposed to high glucose are not further affected by high glucose culture.

Interestingly, primary osteocytes isolated from diabetic rats continued to express elevated sclerostin levels even when cultured under normal glucose conditions. This suggests that diabetes induces long lasting changes to the osteocyte and that high glucose levels are not required for the maintenance of these effects, at least *ex vivo*. Furthermore, osteocytes isolated from control rats did not respond to short-term high glucose treatment by increasing sclerostin and as well, osteocytes isolated from diabetic rats did not decrease sclerostin under short term normal glucose treatment. This indicates that sustained exposure of osteocytes to high glucose is required to induce the elevated sclerostin levels in the diabetic animals. The effects of high glucose levels on *Sost* and sclerostin protein expression are similar *ex vivo* in primary osteocytes and in long term, chronically exposed IDG-SW3 cells (Fig. 8A,B).

One of the most well described functions of sclerostin is as an anti-anabolic factor, inhibiting both bone formation and mineralization by antagonizing the Wnt signaling pathway^31,32^. Increased Sost expression was observed in the tibiae of diabetic mice compared with control mice^33^. However, in contrast with our findings in the tibiae of diabetic rats, no differences were observed in the number of sclerostin positive osteocytes in the ulna of the diabetic or control mice. This could be explained by the different anatomical sites used for the immunohistochemical analysis. The same study has also shown that treatment of diabetic mice with parathyroid-related protein (PTHrP) downregulated sclerostin levels in these animals^33^ and the response to mechanical loading, as evidenced by decrease in sclerostin-positive osteocytes, was blunted in the diabetic animals. PTHrP treatment improved acquisition of bone mass and strength in these mice, suggesting that using PTHrP to regulate sclerostin could be an important therapeutic strategy for maintaining bone health in diabetes.

In addition to negative effects on bone formation, diabetes has also been associated with reduced bone resorption, resulting in low bone turnover^34,35^. Maintenance of bone quality relies upon the removal of old or damaged bone and its replacement. Failure to remove this old or damaged bone, leads to defective bone remodeling and poor bone quality^36,37^. In addition, studies of streptozotocin-induced diabetes in rodents show marked effects on bone, including decreased mineral apposition rate, decreased osteoblastogenesis, and reduction in osteocalcin levels that are similar to spontaneously diabetic mice^38–41^. A decrease in serum carboxylated and non-carboxylated osteocalcin was observed in the diabetic mice compared to controls (Supplemental Data). Osteocalcin, while generally thought to be primarily produced by osteoblasts, is known to be expressed by osteocytes^42^. In addition, Atkins *et al*. showed that sclerostin treatment of late osteoblast/early osteocyte cells *in vitro* significantly down-regulated osteocalcin expression, but had no effect on primary osteoblasts^20^. We consistently saw no effects of elevated glucose on mineral formation *in vitro*, or on expression of osteoblastic genes *in vitro* or *in vivo*.

Altogether, our findings suggest that diabetes induces longstanding changes in osteocytes, leading to upregulation of sclerostin. These effects appear to be mediated by local glucose concentrations and may play an important role in negatively regulating bone quality.

## Supplementary information

Supplementary Figures

Supplementary Figures
