# Supplementary material for: Elevated glucose acts directly on osteocytes to increase sclerostin expression in diabetes
Source: Sci Rep. 2019 Nov 22;9:17353. doi: 10.1038/s41598-019-52224-3 (PMC6874765; doi:10.1038/s41598-019-52224-3)

Elevated glucose acts directly on osteocytes to increase sclerostin expression in diabetes

Donna M Pacicca MD[1,2], Tammy Brown[1], Dara Watkins[1], Karen Kover PhD[1], Yun Yan MD[1], Matthew Prideaux PhD[3] and Lynda Bonewald PhD[3]

[1] Children's Mercy Hospital, Kansas City, Missouri

[2] University of Missouri-Kansas City School of Dentistry, Kansas City, Missouri

[3] Indiana University, Indianapolis, Indiana

Corresponding author:

Donna M Pacicca, MD
Department of Orthopaedic Surgery
Children's Mercy Hospital
2401 Gillham Rd
Kansas City, MO 64108
816-234-3693
dmpacicca@cmh.edu

Supplemental Figure Legends

Supplemental Figure 1. (A) Insulin receptor (*InsR*) expression was unaffected by glucose concentration. (B-F) The expression of osteocyte marker genes *Dmp1*, *E11, Fgf23, RankL, and* OPG was unaffected by glucose.

Supplemental Figure 2. *Rankl* and *Opg* mRNA did not demonstrate significant differences in expression in diabetic vs. control rat cultured cells.

Supplemental Figure 3. Serum carboxylated osteocalcin was significantly decreased in diabetic vs. control rats.

Supplemental Figure 1

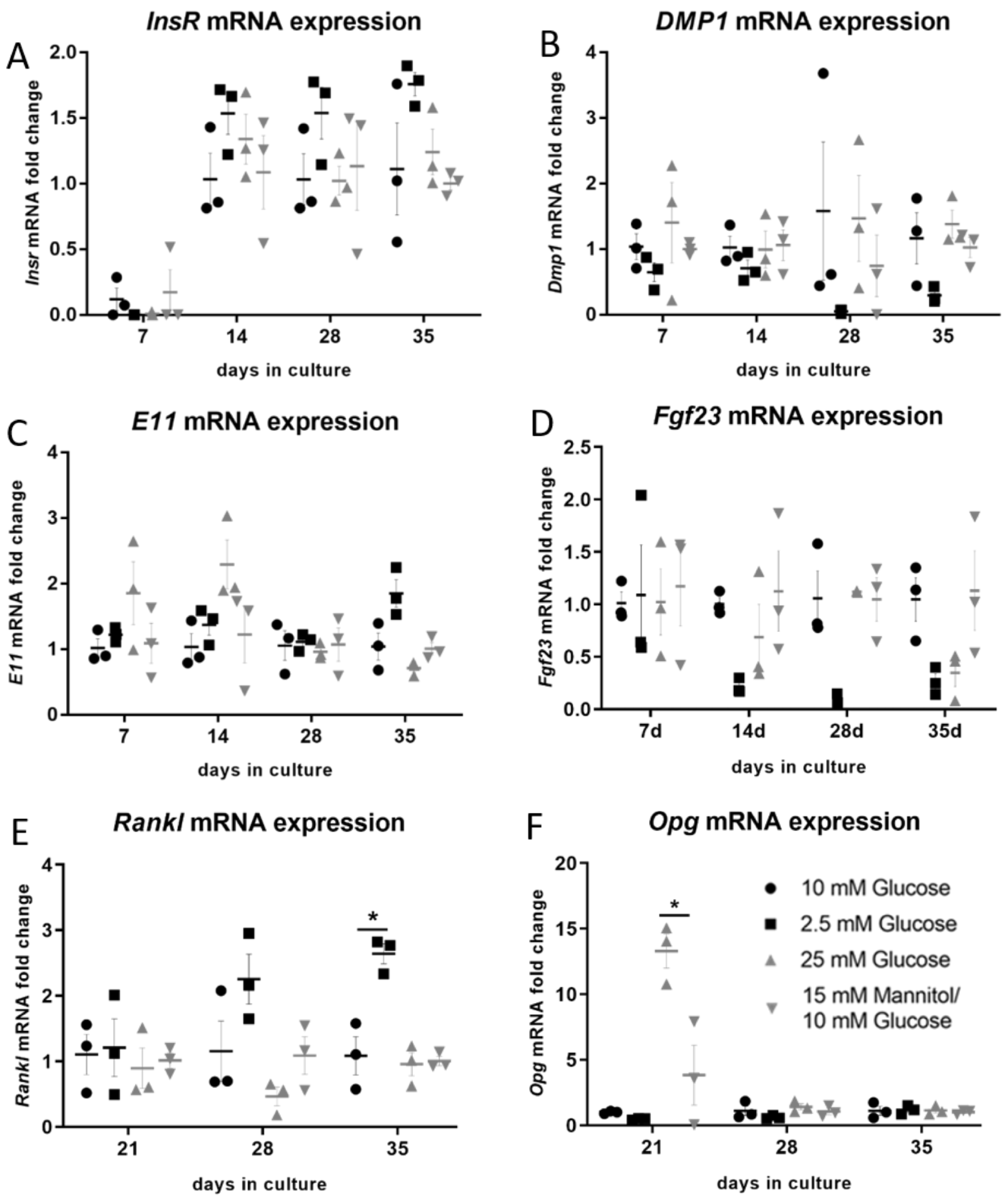

Supplemental Figure 2

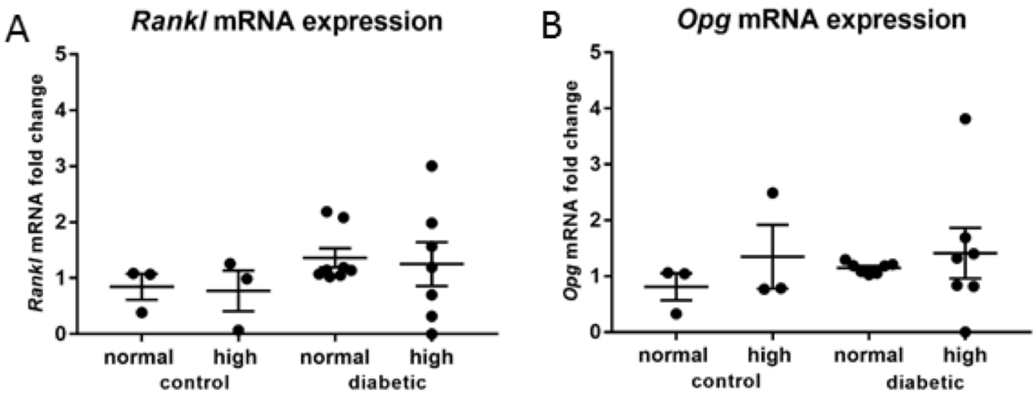

Supplemental Figure 3

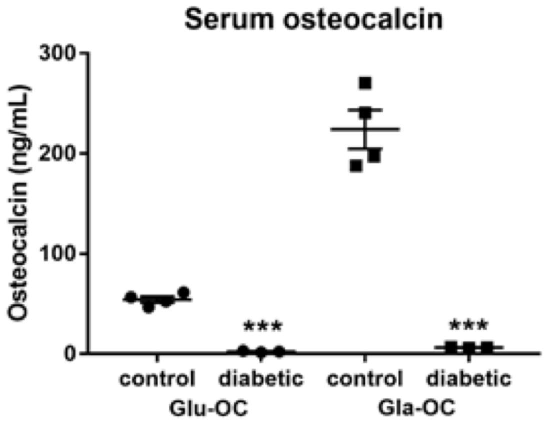

Supplement: Supplementary file 1 — Supplementary Figures [file 41598_2019_52224_MOESM1_ESM.pdf]
