# Supplementary figures and images for: Elevated glucose acts directly on osteocytes to increase sclerostin expression in diabetes
Source: Sci Rep. 2019 Nov 22;9:17353. doi: 10.1038/s41598-019-52224-3 (PMC6874765; doi:10.1038/s41598-019-52224-3)

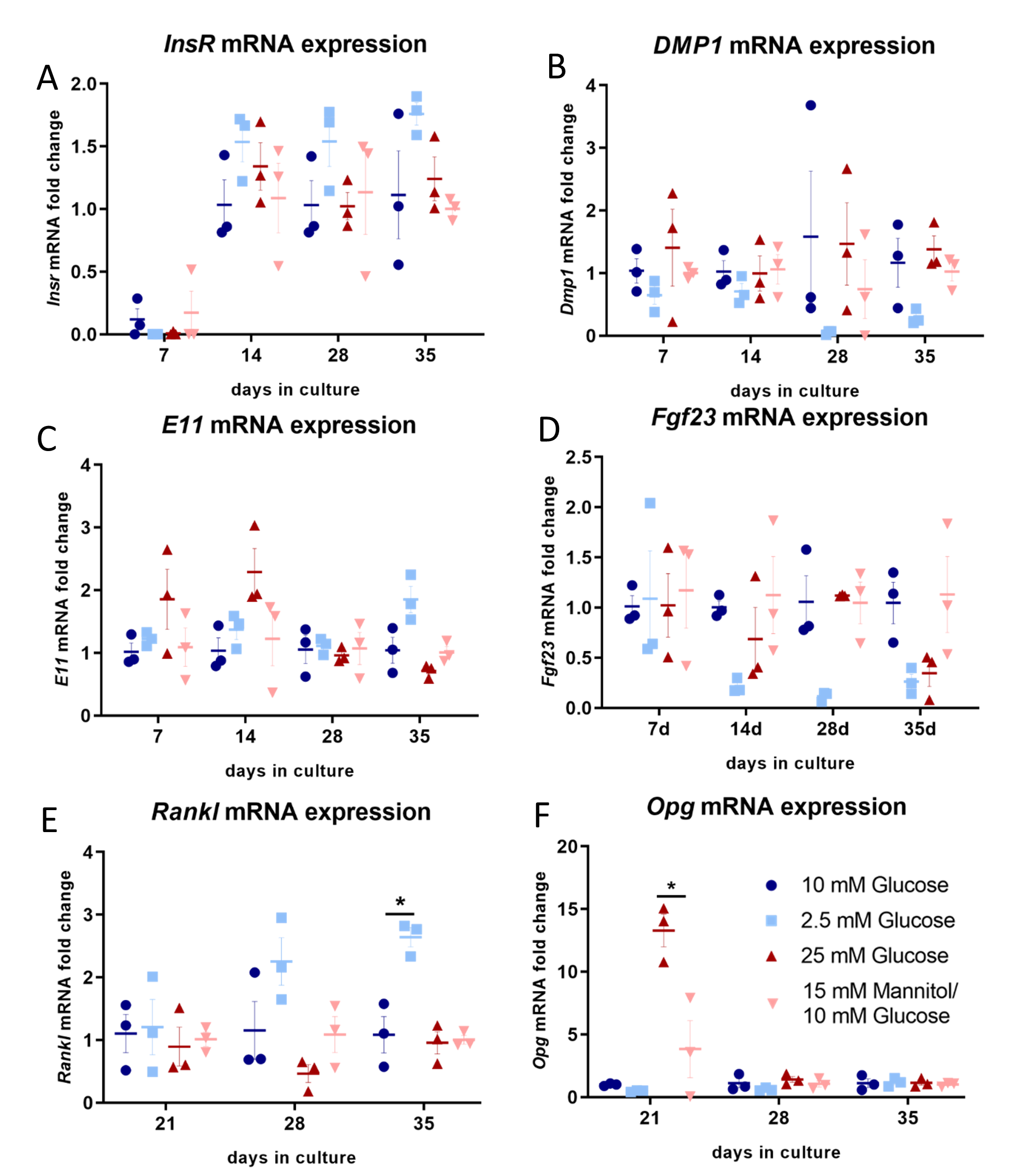

Supplement: Supplementary file 2 — Supplementary Figures [file 41598_2019_52224_MOESM2_ESM.tif]
